# Assessment of balance, sleep quality, and depression in relation to attention in Parkinson’s disease: A cross-sectional P300 study

**DOI:** 10.1590/1516-3180.2026.3465.10032026

**Published:** 2026-07-17

**Authors:** Gulfem Ezgi Ozaltin, Agit Simsek, Busra Candiri, Fatma Ebru Algul, Mehmet Tecellioglu, Burcu Talu

**Affiliations:** I Assistant Professor, Department of Physiotherapy and Rehabilitation, Inonu University (U), Malatya (UF), Türkiye.; II Assistant Professor, Department of Speech and Language Therapy, Inonu University (U), Malatya (UF), Türkiye.; III Assistant Professor, Department of Physiotherapy and Rehabilitation, Inonu University (U), Malatya (UF), Türkiye.; IV Associate Professor, Department of Neurology, Inonu University (U), Malatya (UF), Türkiye.; V Professor, Department of Neurology, Inonu University (U), Malatya (UF), Türkiye.; VI Professor, Department of Physiotherapy and Rehabilitation, Inonu University (U), Malatya (UF), Türkiye.

**Keywords:** Attention, Cognition, Parkinson disease, Postural balance, Neurologic Rehabilitation, Physiotherapy And Rehabilitation, Balance Impairment, Event-related potentials, Sleep disturbances., Cognitive processing

## Abstract

**BACKGROUND:**

Parkinson’s disease (PD) is a progressive neurodegenerative disease characterized by motor and non-motor symptoms. Attention deficits are common in patients with PD, and balance can be affected by sleep quality and depression. The auditory event-related potential (ERP) P300 tool is associated with attention and discrimination processes and is a sensitive indicator of attention.

**OBJECTIVES:**

This study aimed to investigate the effects of balance, sleep quality, and depression on attention in individuals with PD using P300 latency.

**DESIGN AND SETTING:**

In this cross-sectional study, 28 individuals with PD and 27 healthy controls were assessed.

**METHODS:**

Attention was measured using the auditory ERP P300, balance with the Berg Balance Scale (BBS), sleep quality with the Pittsburgh Sleep Quality Index (PSQI), and depression with the Beck Depression Inventory (BDI). Multiple linear regression analysis was used to examine the effects of these variables on P300 latency.

**RESULTS:**

Significant differences were found between the groups in terms of attention, balance, sleep quality, and depression (all P < 0.001). Regression analysis indicated that balance (B = -4.909, P = 0.001) and sleep quality (B = 12.704, P = 0.007) were significantly associated with P300 latency, whereas depression (B = 3.472, P = 0.084) was not.

**CONCLUSION:**

PD is associated with impaired attention, balance deficits, poor sleep quality, and increased depressive symptoms. The significant association between balance performance and sleep quality with attentional processing highlights their potential relevance in clinical assessment and management.

**CLINICAL TRIAL REGISTRATION:**

The study was registered with Clinical Trials (NCT04687371 –  https://clinicaltrials.gov/study/NCT04687371).

## INTRODUCTION

 Parkinson’s disease (PD) is a progressive neurodegenerative disorder characterized primarily by dopaminergic degeneration in the basal ganglia. In addition to motor symptoms, such as tremors, rigidity, gait disturbances, and postural instability, PD is also associated with widespread neurodegeneration affecting the serotonergic, noradrenergic, and cholinergic systems. These changes contribute to a wide range of non-motor symptoms, including cognitive impairment, attention deficits, depression, and sleep disturbances.^1-3^ These non-motor features significantly contribute to disease burden and may interact with motor dysfunction. Cognitive impairment due to subcortical lesion formation is one of the most common symptoms of PD.^3^

 The P300 component of auditory event-related potentials (ERP) is widely used as an objective neurophysiological marker of attentional and cognitive processing. Unlike conventional neuropsychological tests, the P300 provides objective data that are less influenced by motor impairment and patient compliance.^4^ Previous studies investigating P300 latency in patients with PD have reported inconsistent findings. While most studies have demonstrated prolonged P300 latencies, suggesting cognitive slowing, a limited number have reported no significant differences compared to healthy individuals.^5,6^ In addition, the relationships between motor and nonmotor symptoms and attentional processing in patients with PD have generally been examined separately, and their combined associations remain unclear.^7- 9^ Although impairments in attention are well recognized in patients with PD, the clinical factors associated with these changes have not been fully elucidated. In light of this information, we hypothesized that there is a difference in attention levels between the PD and healthy groups, and that dynamic balance, sleep, and depression may be related to attention (P300) in patients with PD. 

## OBJECTIVE

 This study aimed to investigate differences in P300 latency between individuals with PD and healthy controls and to examine the associations of balance, sleep quality, and depressive symptoms with attentional processing in patients with PD. 

## METHODS

### Study Design

 This study was planned as a cross-sectional study. Following institutional and ethics committee approval (Approval No:2022/3749, Date: 25.10.2022), it was conducted in the neurology outpatient clinic of a tertiary research hospital. The study was registered with Clinical Trials. It was conducted in accordance with the principles of the Declaration of Helsinki, and informed consent was obtained from the participants. 

### Sample size and participants

 In the power analysis conducted before the study, it was calculated that at least 27 patients should be included in each group for the change in balance score to be 1 in patients with PD with α = 0.05 and 1-β (power) = 0.80; this calculation was based on balance outcomes from a previous study.^10^

 The study population consisted of patients who applied to the neurology outpatient clinic and were diagnosed with PD by a specialist physician and their relatives. The inclusion criteria for the patients in the study were age between 40 and 85 years, Hoehn and Yahr I, II, or III, literate, and a Mini-Mental State Examination scores > 24. Prior to the P300 assessment, auditory brainstem responses (ABRs) were recorded in all participants. Individuals with a clearly identifiable wave V at stimulus intensities of ≤ 20 dB nHL were considered to have normal hearing and were included in the study, whereas those who did not meet this criterion were excluded. Patients with severe hearing/visual impairment, orthopedic, neurological, or metabolic diseases that could prevent study participation, dementia, those undergoing deep brain stimulation, or those taking anticholinergic, antidepressant, or anxiolytic medications that could affect cognition were excluded. 

### Measurements

 Attention was assessed using a neuro-audio/P300 device. The P300 (P3) wave is a cortical evoked potential that reflects the cognitive process by distinguishing variable stimuli. It was performed with the “oddball paradigm (surprising stimulus sequence)” by randomly placing different stimuli into a standard stimulus sequence. After the patient was in a comfortable sitting position and the bilateral mastoid, forehead region, and area between the two eyebrows were cleaned with gel, reference electrodes (M1, M2) were placed on the bilateral mastoid processes, the positive electrode on the vertex (Cz), and the ground electrode (Fpz) on the forehead region. The patient was then instructed, “Two different sounds will come to your two ears at the same time randomly. One of the sounds will be high-pitched, and the other will be low-pitched. I want you to pay attention to the high-pitched sounds and count them. I want you to report the number to me at the end of the test.” After the information was provided, the headphones were placed bilaterally, and the impedances were checked. P300 latency was calculated as the time from the stimulus onset to the point where the amplitude had a positive or negative peak and was recorded in milliseconds (ms).^4^

 Sleep quality was assessed using the Pittsburgh Sleep Quality Index (PSQI). This is a test that evaluates sleep quality in the preceding month. A maximum of 21 points can be obtained from the test. A score of ≥ 5 indicates poor sleep quality.^11^

 Depression was assessed with the Beck Depression Inventory (BDI). It is a 3-point likert-type scale with 21 items. Higher scores are associated with higher depression severity.^12^

 Balance was assessed using the Berg Balance Scale (BBS). The 14-item scale evaluates balance during sitting, standing, and position changes. The best score is recorded as 56.^13^

### Statistical Analyses

 Data were analyzed using the IBM SPSS Statistics for Windows, version 25.0 (IBM Inc., Armonk, NY, USA). The Shapiro–Wilk test was used to evaluate compliance with the normal distribution. Descriptive statistics are presented as means (standard deviations), frequencies (percentages), medians, and min-max values. An independent sample t-test was used to compare data between groups. Multiple linear regression analysis was used to examine the effects of balance, depression, and sleep quality on the P300, and the P300 was determined as the dependent variable. The statistical significance level of all parameters was accepted as P < 0.05. 

## RESULTS

 We included 55 participants: 28 individuals with PD and 27 healthy controls. The groups did not differ significantly in terms of age, height, weight, sex distribution, educational status, or dominant side (all P > 0.05) (**Table 1**). 

**Table 1:** Comparison of demographic and clinical characteristics between groups

		**Parkinson Group (n = 28)**	**Control Group (n = 27)**	**P**
Age (years)		64.67 ± 9.98	61.55 ± 6.97	0.186^a^
Height (cm)		167.67 ± 8.48	167.59 ± 7.121	0.968^a^
Weight (kg)		78.85 ± 11.08	80.48 ± 8.47	0.545^a^
Sex (Female/Male)		6/22	11/16	0.121^b^
Educational status (primary school/high school/university)		11/12/5	12/11/4	0.914^b^
Time of diagnosis	0–6 months	1		
	6–12months	4		
	1–5 years	23		
Dominant side (Right/Left)		25/3	25/2	0.336^b^
Hoehn and Yahr (I/II/III)		5/15/8		
P300 (ms)		342.42 ± 45.31 (250.70-422.00)	267.99 ± 20.72 (235.60-343.60)	0.001^a^
BBS		32.85 ± 4.45(26-40)	44.07 ± 3.14 (36-52)	0.001^a^
BDI		26.28 ± 1.95(22-30)	12.33 ± 2.14(9-17)	0.001^a^
PSQI		10.85 ± 1.40(8-13)	6.40 ± 1.39(5-9)	0.001^a^

Mean (standard deviation)

a Independent sample t-test

b Chi-Squared test

BBS = Berg Balance Scale; BDI = Beck Depression Inventory; PSQI = Pittsburgh Sleep Quality Index.

**Figure 1:**
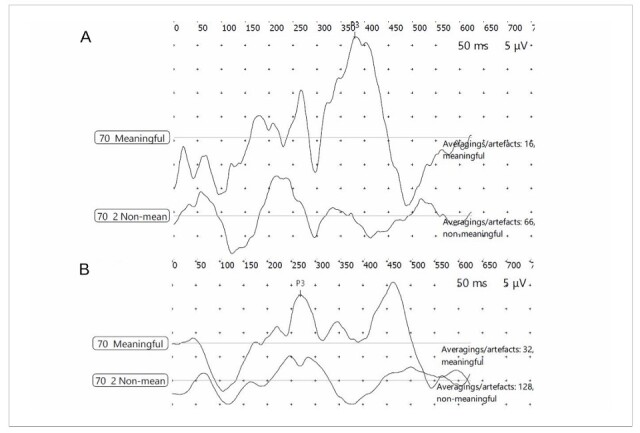
P300 latencies: A. Parkinson’s disease, B. Healthy individual.

 The mean P300 latency was significantly longer in the PD group (342.42 ± 45.31 ms) than in the control group (267.99 ± 20.72 ms, P < 0.001) (**Table 1**). **Figure 1** shows representative P300 waveforms from both groups. 

 BBS scores were significantly lower in the PD group (32.85 ± 4.45) than in the controls (44.07 ± 3.14, P < 0.001). BDI scores were higher in patients with PD (26.28 ± 1.95) than in controls (12.33 ± 2.14, P < 0.001). PSQI scores were also higher in patients with PD (10.85 ± 1.40) than in controls (6.40 ± 1.39, P < 0.001) (**Table 1**). 

 Multiple linear regression analysis revealed that BBS (β = -0.482, P = 0.001) and PSQI (β = 0.394, P = 0.007) scores were significantly associated with P300 latency, together explaining approximately 89% of the variance (adjusted R^2^ = 0.893). Depression (BDI scores) was not significantly associated with P300 latency (P = 0.084) (**Table 2**). Collinearity diagnostics were performed, and the variance inflation factor (VIF) values ranged from 1.74 to 4.45, indicating no severe multicollinearity among the independent variables. 

**Table 2:** Multiple linear regression analysis

	**P300 (adjusted R^2^ = 0.893)**			
	**B**	**S. E.**	**Beta**	**P value**
Constant	274.546	92.887		0.007
BDI	3.472	1.923	0.150	0.084
PSQI	12.704	4.287	0.394	**0.007**
BBS	-4.909	1.294	-.482	**0.001**

BBS = Berg Balance Scale; BDI = Beck Depression Inventory; PSQI = Pittsburgh Sleep Quality Index; SE = Standard Error.

 Sensitivity analyses using separate linear regression models showed that balance performance (R^2^ = 0.837), sleep quality (R^2^ = 0.827), and depressive symptoms (R^2^ = 0.484) were significantly associated with P300 latency. 

## DISCUSSION

 This study demonstrated that P300 latencies were prolonged in individuals with PD compared to healthy controls. In addition, patients with PD exhibited differences in balance, sleep quality, and depressive symptoms. Balance performance and sleep quality were significantly associated with P300 latency, whereas no significant association was observed for depressive symptoms. 

 PD not only causes motor dysfunction but also affects cognitive function, attention, sleep, and depression. P300 is widely used as a neural marker of attentional processing,^4^ and most previous studies have reported prolonged P300 latencies in individuals with PD,^5^ suggesting cognitive slowing. However, a limited number of studies have found no significant differences when compared with healthy controls.^6^ Our findings are consistent with the majority of the literature, demonstrating delayed P300 latency and extending this evidence to individuals with PD without dementia. 

 The relationship between motor and non-motor symptoms in attentional processes has not been fully clarified. In the present study, balance performance was strongly associated with P300 latency. This finding is in line with previous studies, which have demonstrated a close relationship between motor performance, gait, and attention.^14,15^ It may reflect shared neural mechanisms involving frontosubcortical circuits and dopaminergic pathways that contribute to both motor and cognitive functions.^16,17^ Although previous studies have examined the relationship between balance and attention using objective measures, to our knowledge, this study is among the first to investigate this association in Parkinson’s disease by combining dynamic balance assessment with P300 latency analysis. 

 Depression and sleep disorders are common non-motor symptoms in patients with PD. This study showed that there were differences in sleep quality and depression compared to healthy controls. Sleep quality was significantly associated with P300 latency, suggesting that worse sleep may be associated with worse attentional processing in patients with PD. In contrast, depressive symptoms demonstrated a more moderate association with P300 latency. However, this finding should be interpreted with caution. The borderline P value (P = 0.084) and the relatively small sample size suggest that the study may have been underpowered to detect a potential association, and a type II error cannot be excluded. Therefore, the absence of a statistically significant association does not necessarily indicate the absence of a true relationship. Previous studies have reported inconsistent findings regarding the relationship between depression and cognitive function in Parkinson’s disease.^4,8,20^ For example, Norman et al. reported that while patients with depression showed worse performance in memory domains, no differences were observed in other cognitive functions. These inconsistencies suggest that the relationship between depression and cognition in PD is complex and may be influenced by multiple clinical and neurobiological factors.^18^ In addition, the heterogeneous nature of depression, the presence of multiple contributing factors, and the underlying pathophysiology of PD may have contributed to the lack of a clear association observed in this study. 

 Other non-motor symptoms affecting the quality of life in patients with PD are sleep disorders. Impaired serotonin and dopaminergic systems also lead to sleep difficulties. It can be suggested that there is an association between sleep disorders and attention in patients with PD.^19^ However, more comprehensive studies are needed to understand whether this situation is due to the pathology of the disease or only to changes in cognitive function. Stavitski et al. investigated the relationship between sleep quality and cognitive function in PD and showed that poor sleep is associated with attention and executive function.^20^ The basal ganglia and cortical structures project to the hypothalamus, which regulates sleep-wake regulation.^21^ This may explain sleep disturbance in patients with PD. The relationship between attention levels and sleep may be due to some common pathological pathways, such as the involvement of the mesolimbic dopamine circuit that occurs with the disease.^20^ Latreille et al. demonstrated that sleep spindle density can be used as a potential biomarker of cognitive function.^22^

 Given the strong interrelationships among balance performance, sleep quality, and depressive symptoms, the observed multicollinearity likely reflects overlapping clinical and neurobiological mechanisms inherent to PD. Rather than indicating statistical instability, this interrelatedness highlights the complex interaction between motor and non-motor symptoms influencing attentional processing, as reflected by P300 latency. 

 This study has several limitations that should be considered when interpreting the findings. First, the relatively small sample size may limit the stability of the regression coefficients and the generalizability of the findings. Second, the presence of multicollinearity among balance performance, sleep quality, and depressive symptoms restricts the interpretation of fully independent effects in multiple regression analyses. However, this interrelatedness likely reflects overlapping clinical and neurobiological mechanisms inherent to PD rather than a statistical artifact. To address this issue, sensitivity analyses using separate regression models were performed, which confirmed the robustness of the associations between P300 latency and balance performance, sleep quality, and depressive symptoms. Third, although disease duration and Hoehn and Yahr stages were recorded, they were not included in the regression analysis. In addition, PD-specific clinical variables, such as medication status (e.g., levodopa dosage and ON/OFF state), and non-motor symptoms, including fatigue, anxiety, and apathy, were not systematically assessed. Owing to the relatively small sample size, the number of predictors included in the regression model was intentionally limited to avoid overfitting and instability of the estimates. The absence of these covariates may have confounded the observed associations and should be considered when interpreting the results. Future studies with larger sample sizes and longitudinal designs are warranted to further clarify the relative and potentially causal contributions of motor and non motor symptoms to attentional processing in PD. 

## CONCLUSION

 This study demonstrated prolonged P300 latency in individuals with PD, along with differences in balance, sleep quality, and depressive symptoms compared to healthy controls. The findings further indicate that balance performance and sleep quality are significantly associated with attentional processing in PD. To our knowledge, this is one of the first studies to examine the combined associations of motor and non-motor features with attention using P300 measures in PD. These findings contribute to a better understanding of the complex interplay between motor and non-motor symptoms and their relationship with attentional processing in PD. However, the findings regarding depression should be interpreted with caution, as the lack of a significant association may be related to limited statistical power. Future studies with larger sample sizes and longitudinal designs are needed to further clarify these relationships. 

## Data Availability

The data supporting the findings of this study are available from the corresponding author upon reasonable request. Owing to ethical considerations, the data are not publicly available
